# Reduced Oxygen Condition Is Associated with Genome-Wide Expression Changes in Mastitis-Lineage *Staphylococcus aureus* During In Vitro Invasion into a Mammary Cell Line

**DOI:** 10.3390/ijms27104591

**Published:** 2026-05-20

**Authors:** Kamaleldin B. Said, Marcus B. Jones, Rosslyn Maybank, Scott N. Peterson, Xin Zhao

**Affiliations:** 1 Department of Pathology, College of Medicine, University of Ha’il, Ha’il 55473, Saudi Arabia; 2 Regeneron Pharmaceuticals, Tarrytown, NY 10591, USA; 3 Walter Reed Army Institute of Research, Silver Spring, MD 20910, USA; 4 Sanford Burnham Prebys, La Jolla, CA 92037, USA; 5 Department of Animal Science, McGill University, 21111 Lakeshore Road, Sainte-Anne-de-Bellevue, QC H9X 3V9, Canada

**Keywords:** bovine mastitis, intramammary *Staphylococcus aureus*, mammary epithelial cells, reduced oxygen, genome-wide expression

## Abstract

*Staphylococcus aureus* (*S. aureus*) bovine mastitis is a significant public health issue. Despite enormous efforts, important gaps remain regarding host–microenvironmental factors. How intramammary reduced oxygen modulates *S. aureus* transcription in bovine mammary epithelial cells (MECs) remains unclear. We examined oxygen-associated transcriptional changes in a bovine-mammary adapted *S. aureus* clone following internalization into MECs and identified functional category enrichments under Normal-O_2_ and Reduced-O_2_ exposures. Bovine MAC-T monolayers were infected with a dominant bovine mastitis isolate under Normal-O_2_ or Reduced-O_2_ conditions. Triplicate infection experiments were performed for each oxygen condition. Each condition included matched non-reacted bacterial controls maintained under the same gas condition but without MAC-T exposure serving as the reference condition for expression calling. RNA was extracted and profiled using a high-throughput qRT-PCR platform covering genome-wide loci. Expression calls were mapped to curated BioQT roles and interpreted descriptively. Results indicated 211 loci were upregulated and 99 were downregulated under Normal-O_2_ conditions, versus 53 upregulated and 35 downregulated under Reduced-O_2_ conditions, relative to their non-reacted controls. Under Normal-O_2_ conditions, regulated loci covered multiple functional roles, including cellular processes, transport/binding proteins, regulatory functions, and energy metabolism with downregulated loci enriched in transport/binding and cell-envelope categories. Under Reduced-O_2_ conditions, upregulated loci were abundant in cellular process annotations dominated by pathogenesis/toxin-related functions, whereas downregulated loci were enriched in nucleotide biosynthetic and DNA/cell division categories. Thus, this reveals oxygen-associated shifts in the transcriptional response of intramammary *S. aureus* in MAC-T cells. Normal-O_2_ conditions were associated with broader category representation, whereas Reduced-O_2_ conditions yielded a narrower distribution enriched for selected toxin/pathogenesis- and iron/cation-associated annotations. These oxygen-linked transcriptional-shifts highlight candidate pathways for the intramammary adaptation of *S. aureus*, potential diagnostic markers, anti-virulence strategies, and targeted therapeutics.

## 1. Introduction

*Staphylococcus aureus* (*S. aureus*) is a leading cause of bovine intramammary infection (IMI), driving persistent, difficult-to-cure mastitis that erodes milk yield, compromises animal welfare, and imposes substantial economic losses on dairy systems worldwide. Recent syntheses estimate multi-billion-dollar global impacts with subclinical, recurrent infections contributing disproportionately to hidden costs and culling risk [1,2,3]. Beyond farm economics, bovine *S. aureus* has zoonotic relevance with livestock-adapted lineages periodically breaching species barriers [4].

Population-genomic work over the last decade shows that bovine disease is dominated by a restricted set of clonal complexes (CCs)—notably CC97 and CC151—whose accessory genomes encode distinct complements of surface adhesins, toxins, and iron-acquisition systems aligned with ruminant tissues [5,6,7]. These bovine-adapted lineages differ in adhesion, invasion, and biofilm traits, helping to explain herd-to-herd variability in clinical course and treatment response [8]. Importantly, *S. aureus* is not confined to the milk compartment: it adheres to and invades mammary epithelial cells (MECs), where it can persist intracellularly, evade humoral defenses, and tolerate antibiotics—mechanistic features strongly linked to chronic and relapsing mastitis [9,10]. Comparative infection studies in MAC-T bovine mammary epithelial cells (MAC-Ts) corroborate strain-dependent differences in internalization and cytotoxicity, strengthening the case that host–cell niches and bacterial background co-determine disease biology [11].

A central—but underexplored—determinant of this intracellular niche is oxygen availability. Oxygen limitation is a recognized feature of several infection microenvironments and is known to influence *S. aureus* physiology, stress adaptation, metabolism, and virulence factor regulation [12,13,14]. *S. aureus* encodes dedicated sensory circuits proteins for low-oxygen and nitrosative stress, most prominently the SrrAB two-component system, which rewires metabolism, respiration, and toxin expression to sustain growth under oxygen limitation and host immune pressure [11,13,15]. In parallel, iron restriction is a defining property of mammalian tissues. To overcome nutritional immunity, *S. aureus* deploys the iron-regulated surface determinant protein (Isd) apparatus and siderophore systems; recent work highlights that efficient heme capture requires coordinated cell-envelope architecture [16,17]. Because oxygen tension and iron availability are tightly coupled in vivo, these inputs likely operate together to gate the transition between acute damage (toxin-rich) signatures and persistence (low-metabolic) states during mammary infection.

Despite this framework, most transcriptional studies informing oxygen-responsive virulence have focused on human clinical isolates, planktonic cultures, or biofilms grown in fermenters. Direct, organ-relevant measurements of oxygen-conditioned gene expression in bovine-adapted strains during their intracellular phase in mammary epithelium remain scarce. MEC invasion has been exploited to probe anti-invasion strategies and alternative therapeutics (e.g., bacteriophage), underscoring clinical urgency, but these experiments rarely resolve global regulatory wiring under defined oxygen regimes [18,19]. Likewise, while lineage surveys catalog virulence gene carriage across bovine CCs, carriage is a poor surrogate for context-specific expression, and it cannot predict how the mammary microenvironment routes signals through global regulators (Agr/Sar family, SrrAB) to deploy toxins, adhesins, or metabolic circuits at the right time and place [8]. Finally, intracellular persistence and small-colony-variant (SCV) phenotypes—hallmarks of chronic mastitis—are intimately tied to altered respiration and carbon flux, yet how bovine mammary oxygen levels program these metabolic–virulence couplings has not been systematically mapped [20,21].

These gaps are not merely academic. Oxygen- and iron-sensitive regulons integrate signals that are actionable for control: they can expose vulnerabilities to anti-virulence or anti-metal strategies, inform diagnostics that read out organ-specific transcriptional states, and refine typing schemes beyond static allele calls to functional expressions [22,23]. Moreover, a transcript-level view within the mammary epithelial niche is essential to reconcile divergent observations from fermenter studies and to prioritize targets whose expression is actually engaged during intracellular residence in bovine tissue.

Accordingly, this paper focuses on bovine-adapted *S. aureus* internalized into bovine MECs and asks a specific question: how does oxygen availability within the mammary epithelial niche shape genome-wide transcriptional patterns related to adhesion, toxin production, nutrient acquisition, including heme/iron uptake, and metabolic adaptation?

To answer this, we profile whole-genome expression after host-cell internalization under defined “normal” and “reduced” oxygen conditions using a high-throughput qRT-PCR platform spanning multiple reference genomes. This organ- and condition-matched approach is designed to (i) resolve oxygen-conditioned activation of major global regulators and effector modules relevant to bovine disease; (ii) distinguish active virulence-oriented versus persistence-oriented circuits in the intracellular niche; and (iii) nominate tractable, organ-specific markers that could improve detection, prevention, and targeted therapeutics for bovine mastitis. By situating transcriptional control in the appropriate host cell and physiologic oxygen range—and by leveraging bovine-adapted lineages—this paper aims to close a critical knowledge gap between gene carriage and context-dependent expression in the pathogenesis of intramammary *S. aureus*.

## 2. Results

### 2.1. Analytical Approach and Primary Readouts

**Figure 1:**
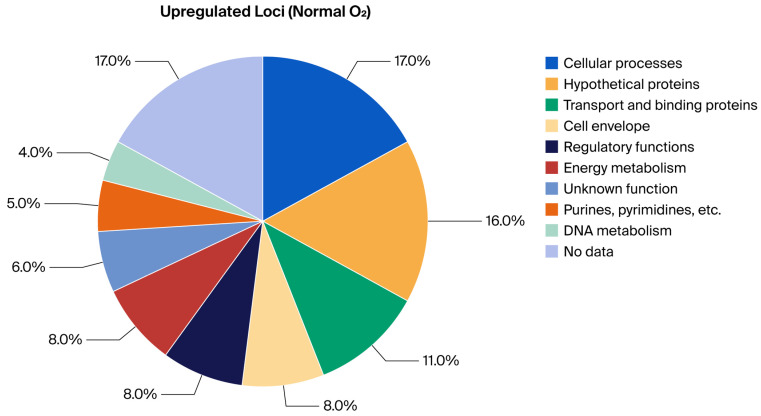
Visual summary of BioQT main biological role distribution among *S. aureus* loci upregulated under Normal O_2_ relative to the matched non-reacted Normal O_2_ bacterial control (*n* = 211); values are within-condition percentages.

**Figure 2:**
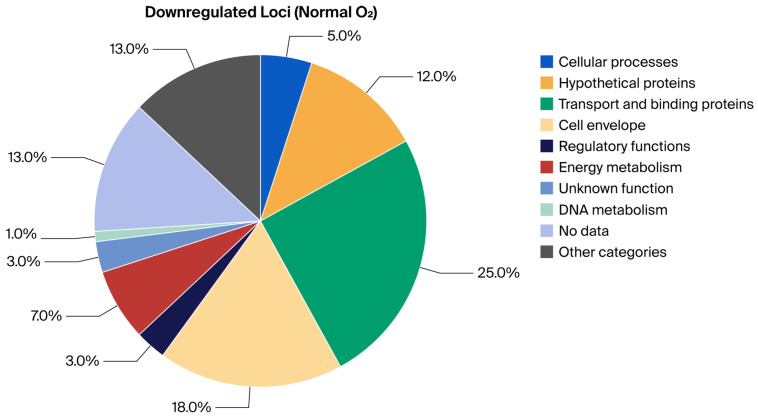
Visual summary of BioQT main biological role distribution among loci downregulated under Normal O_2_ relative to the matched non-reacted Normal O_2_ bacterial control (*n* = 99); values are within-condition percentages.

**Figure 3:**
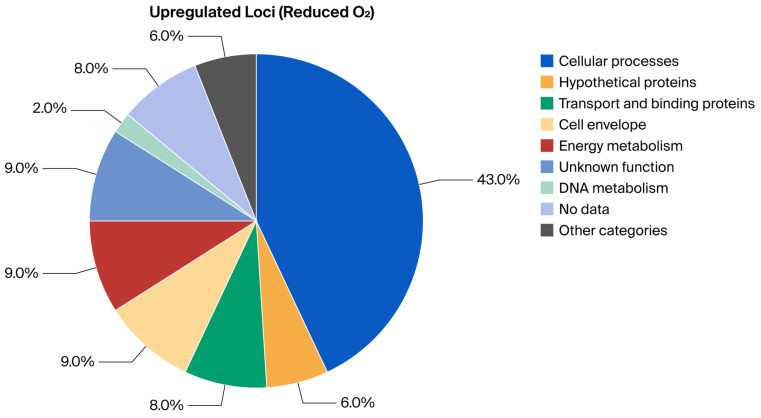
Visual summary of BioQT main biological role distribution among loci upregulated under Reduced O_2_ relative to the matched non-reacted Reduced O_2_ bacterial control (*n* = 53); values are within-condition percentages.

**Figure 4:**
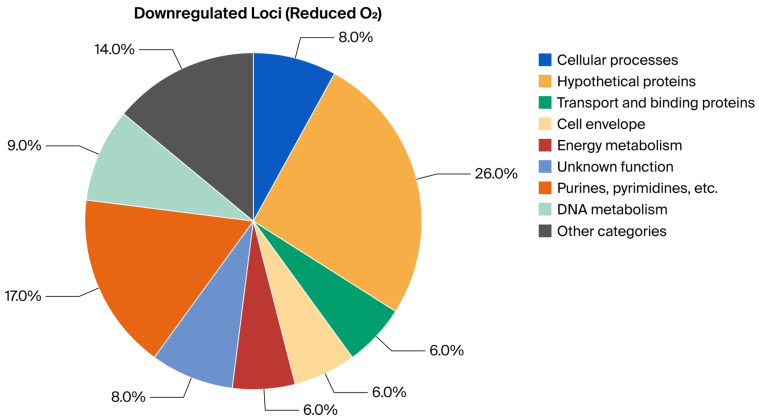
Visual summary of BioQT main biological role distribution among loci downregulated under Reduced O_2_ relative to the matched non-reacted Reduced O_2_ bacterial control (*n* = 35); values are within-condition percentages.

To investigate oxygen-dependent transcriptional responses, locus-expression profiles were first normalized within the high-throughput qRT-PCR workflow and then classified relative to matched non-reacted bacterial controls maintained under the same oxygen condition. These non-reacted controls were bacteria processed under the same gas-phase condition but without MAC-T cell exposure; they were not housekeeping genes and were not used as target-gene comparators. The key findings are summarized in Table 1, Table 2, Table 3 and Table 4 and Figure 1, Figure 2, Figure 3 and Figure 4, which report functional-category distributions and selected representative loci. Complete condition-specific inventories of regulated loci are provided for upregulated genes at Normal O_2_ (Supplementary Table S1), downregulated genes at Normal O_2_ (Supplementary Table S2), upregulated genes at Reduced O_2_ (Supplementary Table S3), and downregulated genes at Reduced O_2_ (Supplementary Table S4).

### 2.2. Overview of Oxygen-Dependent Transcriptional Responses

Across the four expression-call sets and three independent biological replicate experiments (*n* = 3), the same matched-control Cp-difference workflow was applied to both oxygen conditions. A total of 211 loci were classified as upregulated and 99 as downregulated under Normal O_2_ relative to the matched non-reacted Normal O_2_ bacterial control. In contrast, 53 loci were classified as upregulated and 35 as downregulated under Reduced O_2_ relative to the matched non-reacted Reduced O_2_ bacterial control. These counts indicate that in this dataset, the regulated-locus set under Normal O_2_ was broader than the regulated-locus set under Reduced O_2_. The comparison between oxygen conditions is therefore presented as a comparison of matched-control-corrected expression-call sets and their functional distributions, not as a housekeeping-gene-normalized direct gene-by-gene ΔΔCp model.

The functional distributions highlight oxygen-associated differences in matched-control-corrected expression-call sets. Under Normal O_2_, upregulated genes were broadly distributed across categories with notable contributions from Cellular Processes (17.5%), Hypothetical Proteins (16.6%), and Transport and Binding Proteins (11.4%). Downregulation was more targeted, being dominated by Transport and Binding Proteins (24.2%) and Cell Envelope functions (18.2%), suggesting a specific remodeling of the bacterial surface (Table 1; Figure 1 and Figure 2; Supplementary Tables S1 and S2).

The response to Reduced O_2_ was more concentrated within fewer functional categories when analyzed using the same matched-control criteria. Upregulated genes were heavily skewed toward Cellular Processes, which accounted for 43.4% of the total and were dominated by pathogenesis/toxin-related annotations. Downregulated genes under Reduced O_2_ were comparatively enriched for nucleotide metabolism, DNA-associated functions, and cell-division-related categories, which was consistent with a narrower transcriptional profile in this condition (Table 2; Figure 3 and Figure 4; Supplementary Tables S3 and S4).

### 2.3. Key Transcriptional Signatures

The analysis of individual genes identified a shared set of toxin- and virulence-associated loci represented under both oxygen conditions alongside condition-associated gene subsets (Table 3). A recurring group included loci encoding *alpha-hemolysin* (*hly*), *delta-hemolysin* (*hld*), *phenol-soluble modulins*, *leukocidins*, and *sspB*. In Table 3, “upregulated” and “downregulated” mean that the locus met the predefined >2 Cp-cycle threshold, corresponding to an approximately >4-fold difference in the indicated direction relative to the matched non-reacted bacterial control for that same oxygen condition. “Not significant” indicates that this fold-difference criterion and/or the duplicate-variance criterion was not met. Beyond this shared set, the distribution of regulated loci differed by oxygen condition and is therefore presented as an oxygen-associated transcriptional pattern rather than as proof of a distinct biological program.

However, the patterns of regulated loci differed beyond this core. Under Normal O_2_, the response was characterized by broader regulation across multiple functional categories (Table 1) with a prominent representation of regulatory functions and transport and binding proteins. Consistent with this, several regulatory loci were upregulated, including the *agr quorum-sensing system* (*agrD*), the staphylococcal accessory regulator (sarU), and two-component systems linked to cell-envelope stress sensing (vraS, yycG). This was accompanied by the induction of numerous transport systems, including a large array of ABC transporters for amino acids/peptides and permeases for nucleosides (Supplementary Table S1). In addition, multiple loci annotated to energy metabolism were increased under Normal O_2_, including enzymes consistent with fermentative/redox-associated carbon flux (e.g., *ldh1*, *pflA/B*, *fdaB*) (Supplementary Table S1).

In contrast, under reduced oxygen, the regulated loci were more concentrated in specific functional categories (Table 2) with a prominent representation of pathogenesis/toxin-associated annotations and iron acquisition functions. For example, *S. aureus* showed an increased expression of genes encoding iron-regulated surface determinant proteins, including *isdA* and *isdC* (Supplementary Table S3). This was complemented by the upregulation of adhesins, including a key fibrinogen-binding protein (SACOL1220). Compared with normoxia, this condition showed less induction of global regulators and diverse transport systems, indicating a narrower set of regulated categories under Reduced O_2_ that is consistent with adaptation to a more restrictive intracellular environment without implying functional confirmation of a discrete biological phenotype.

The downregulation patterns further supported oxygen-associated differences in functional category distribution. Under normoxia, downregulation was largely targeted at the cell surface with a notable metabolic change being the suppression of lactate dehydrogenase 2 (*ldh2*) (Supplementary Table S2). Conversely, Reduced O_2_ was associated with the suppression of core biosynthetic and cell-replication machinery. This included the consistent downregulation of genes encoding products involved in pyrimidine biosynthesis (e.g., *pyrC*, *pyrF*, *carA/B*) and genes encoding products required for cell division (*ftsZ*) and cell wall synthesis (*mraY*, *murD*) (Table 3, Supplementary Table S4). This pattern is consistent with reduced biosynthetic and replication-associated activity under the tested condition, but it should not be interpreted as direct proof of persistence or dormancy without additional functional assays.

### 2.4. Shared and Condition-Specific Gene Regulation

An analysis of the overlap between gene sets highlights shared and condition-associated components of the oxygen response (Table 4). Fourteen loci were classified as upregulated in both conditions, including *hly*, *leukocidin*-associated loci, and *sspB*. Beyond this shared set, 161 loci were classified as upregulated only under Normal O_2_, and 19 were classified as upregulated only under Reduced O_2_. Because the analysis is based on matched-control-corrected expression calls from three independent experiments, these overlaps are presented descriptively rather than as a direct measure of functional hierarchy.

## 3. Discussion

This paper shows that oxygen availability is associated with distinct transcriptional profiles in intracellular *S. aureus* recovered from MAC-T bovine mammary epithelial cells. Within this broader oxygen-associated response, one of the most consistent findings was the recurring representation of toxin-associated transcripts under both oxygen conditions. The persistence of α-hemolysin (*Hla*) across conditions is biologically plausible: Hla is essential for tissue injury in multiple infection models and drives epithelial and endothelial damage, thereby facilitating spread and nutrient access. In murine pneumonia, Hla is required for full virulence; in skin and soft-tissue infection models, it damages diverse host cells and accelerates pathology [24,25]. Importantly, the oxygen terms in this paper refer to the defined gas-phase exposure conditions used during the MAC-T infection assay. Accordingly, references to ‘persistence’, ‘dormancy,’ or ‘intracellular survival’ are presented as cautious inferences from transcriptional patterns rather than as directly measured phenotypes.

Under Normal O_2_, the dataset shows broader transcriptional representation with an upregulation of transporters and transcriptional regulators that could expand the metabolic range and coordinate adaptation inside host cells. This interpretation remains conservative: the data indicate enriched functional categories and selected loci, but they do not by themselves establish a complete mechanistic program.

The regulatory landscape accompanying this broad activation includes the two-component systems and global regulators that integrate environmental cues with virulence. The upregulation of systems like VraSR, a sentinel of cell-wall stress, is strongly inducible by peptidoglycan inhibitors and coordinates a protective stimulon; its activation intersects with the envelope remodeling often seen during intracellular growth [26,27]. WalKR (YycG/YycF), the essential cell-wall TCS, orchestrates autolysis, cell-wall metabolism and broad cellular signatures, and it has been mechanistically linked to growth control; its activity readily interfaces with other regulators in normoxic growth [28,29]. MgrA, a pleiotropic regulator, shapes adhesion, capsule, and exoprotein expression and measurably affects invasion phenotypes—again aligning with a normoxic, regulator-rich state [30,31]. Finally, *agr* expression itself ties metabolism to virulence and possesses an oxidation-sensing input; the normoxic enhancement of quorum signaling can increase cytotoxin expression while modulating surface factors [32,33]. These regulatory axes provide a mechanistic basis for why, under normoxia, we observed broad transporter/regulator signatures coexisting with classical toxins.

Under Reduced O_2_, the upregulated loci encoded products concentrated in selected functional categories rather than broadly distributed. The transcriptional pattern includes effectors for iron acquisition and surface interaction. The *Isd* system is central here: *IsdA* promotes survival on skin and nasal colonization, and direct heme transfer from IsdC contributes to iron capture from host hemoproteins [34,35,36]. Under Reduced O_2_, the regulated loci remain enriched for toxin/pathogenesis-related annotations, which is consistent with a toxin-skewed transcriptional profile. The *agr*-controlled cytolysins noted above remain prevalent, and mounting evidence indicates that PSMs and leukocidins facilitate intracellular survival and egress by killing phagocytes and damaging non-professional phagocytes, which is a strategy that may be favored when oxidative phosphorylation is constrained [37]. The hypoxic imprint on *S. aureus* gene regulation is well documented: the SrrAB two-component system senses electron-transport flux and nitric oxide, reprogramming metabolism and virulence during oxygen limitation, while NreABC controls nitrate/nitrite respiration and links redox to anaerobic gene expression [13,38,39]. Recent work demonstrates SrrAB-dependent shifts in biofilm and stress responses when respiration is impaired, providing a regulatory scaffold that is consistent with the toxin/iron/cation signature we observe under reduced oxygen [39].

Across both oxygen regimens, we detect signatures of central metabolic reprioritization. The presence of *ldh1* with other fermentative enzymes under normoxia is not paradoxical: *ldh1* is inducible by nitric oxide and glucose and is indispensable for maintaining redox balance during host-derived nitrosative stress; this signature supports acute survival even when O_2_ is available, echoing the intracellular milieu [40,41]. Pyruvate-formate lyase (Pfl) and its activating enzyme contribute under oxygen-limited states by supplying formate and acetyl-CoA; genetic perturbations of Pfl/Fdh rewire anaerobic metabolism and impact fitness, which is consistent with our detection of Pfl-linked loci [42]. Our data also show that succinate dehydrogenase (SdhC) is upregulated under reduced oxygen, which is a metabolic adaptation that benefits biofilm growth in nutrient/oxygen gradients typical of intracellular microenvironments [43]. This metabolic plasticity has direct immunologic consequences: *S. aureus* lactate production raises IL-10 via HDAC6 activity in myeloid cells, dampening antibacterial responses and fostering persistence [44].

The significant induction of various transporters for ions and solutes highlights the importance of osmoadaptation as a metabolic–environmental coupling. The Ktr system and NaCl-responsive regulons are critical for growth across ionic stresses and are coordinately regulated with multiple transporters we see induced, providing a plausible route by which cells in the intracellular niche strengthen uptake capacity [44,45]. This point is particularly relevant because *S. aureus* osmoadaptation is not limited to inorganic ion transport. Compatible-solute uptake, especially proline and glycine betaine transport, is a well-established strategy by which *S. aureus* preserves intracellular water balance, protein stability, and growth under osmotic stress. Earlier work showed that L-proline enhances *S. aureus* growth in a high-osmolarity medium and is taken up through high- and low-affinity transport systems, supporting its role as an osmoprotectant. Glycine betaine is also accumulated by *S. aureus* under low-water-activity conditions through dedicated uptake systems, and broader osmoprotection studies identify proline, glycine betaine, choline, and related compatible solutes as major contributors to staphylococcal osmotolerance. Therefore, the transporter-rich signature observed here may reflect not only ion handling through Ktr/Kdp-related systems but also a broader compatible-solute response that could help intracellular bacteria tolerate osmotic and metabolic stress during epithelial-cell residence [46,47,48].

The functional configuration we observe is consonant with studies in bovine mammary epithelial cells (MAC-Ts) and other epithelial models. *S. aureus* invades and persists within MAC-T cells, surviving intracellularly and escaping via cytolysin-mediated damage; this phenotype was recognized in early MAC-T work and is echoed in more recent epithelial systems [49]. Intracellular survival is aided by toxins and proteases; for example, a bacterial cysteine protease has been shown to drive host cell death during intracellular residence, and PSMs potentiate the lysis of infected non-professional phagocytes and osteoblasts, promoting bacterial release [37,50]. In MAC-T and mammary models, α-toxin specifically injures gland epithelium and disrupts barrier function, which is consistent with the strong representation of Hla across our conditions [51,52]. Mammary epithelial infections also generate localized hypoxia, and SrrAB-linked rewiring under hypoxic stress alters bacterial virulence and host responses—again aligning with the dominance of toxins and iron capture we see when oxygen is reduced [53].

Mechanistically, our patterns can be interpreted through interacting regulatory layers. *agr* integrates quorum and oxidative inputs, directly controlling PSM genes through AgrA in an RNAIII-independent manner, while RNAIII modulates a broader exoprotein program that includes hemolysins, proteases, and surface-associated factors [33,54]. Thus, *agr* may help explain why cytolysin-, PSM-, leukocidin-, and protease-associated loci remained represented across both oxygen conditions even as the metabolic and transport categories differed. Under conditions that favor respiration, WalKR and VraSR coordinate envelope remodeling and growth, while MgrA calibrates the balance between surface adhesins and exoproteins, including effects on invasion and capsule, mirroring the broad regulator/transporter emphasis observed under Normal O_2_ [27,28]. When oxygen becomes limiting, SrrAB and NreABC re-prioritize anaerobic respiration, stress endurance, cytotoxin output, and biofilm-associated traits [13,14]. This same regulatory network intersects with *nos* and redox-stress pathways, including PerR, which can influence leukocidin expression and neutrophil cytotoxicity [55,56]. Together, these findings support a conservative model in which Agr acts alongside oxygen-responsive and redox-stress regulators: Agr may help maintain cytolysin and exoprotein expression, while SrrAB/NreABC and related pathways reshape respiration, stress tolerance, and transport under reduced oxygen.

Independent in vitro and ex vivo datasets reinforce the dual oxygen signature indicated by our analysis. Transcriptome studies under osmotic or ionic stress reveal large transporter regulons, including ABC and permease systems, while MAC-T and bovine mammary infection models document epithelial cytokine responses coincident with staphylococcal pore-forming toxins [46,52]. An RNA-seq of infected mammary epithelial cells confirms the extensive remodeling of host–pathogen interfaces, which is consistent with the need for bacterial transport/regulatory modules under normoxia and cytolysin-driven escape strategies under oxygen limitation [57]. At the same time, genome-scale and mechanistic studies of SrrAB demonstrate an oxygen-responsive re-wiring of virulence that dovetails with our hypoxia-skewed toxin/iron profile [39].

This paper provides a technically supported genome-wide qRT-PCR profile of intracellular *S. aureus* transcription under defined Normal O_2_ and Reduced O_2_ exposure conditions. The use of matched non-reacted bacterial controls, biological triplicates, technical duplicates, plate-level mean-centering, and predefined Cp-difference thresholds supports the robustness of the locus-level expression calls. Importantly, housekeeping genes were not used as comparator outputs for genes of interest; instead, the matched non-reacted bacterial controls served as the reference condition for expression calling within each oxygen condition. Although intracellular bacterial burden, host-cell ROS, and bacterial subcellular localization were not directly quantified, the observed patterns identify oxygen-linked transcriptional modules involving toxin/pathogenesis, iron/cation acquisition, transport, regulation, and metabolic adaptation. These findings provide a focused framework for future validation studies and may help prioritize diagnostic markers, anti-virulence targets, and oxygen-context-dependent therapeutic strategies for bovine mastitis-associated *S. aureus*.

Two practical implications follow. First, the shared toxin/PSM/leukocidin core across oxygen states argues that anti-virulence strategies against Hla, PSMα, and leukocidins could blunt disease regardless of microenvironmental oxygenation. The centrality of these effectors is supported by genetic and pharmacologic attenuation studies across models [24,58,59]. Second, oxygen-conditioned adjuncts may be rational: inhibitors of iron capture or cation transport could be most impactful where oxygen is low and Isd/cation systems are prominent, while transport/regulator targeting (e.g., Opp3/DtpT nutrient gating or MgrA/VraSR tuning) might weaken normoxia-favored signatures [27,30,60]. Finally, the metabolic nodes we observe—Ldh1 and Pfl—represent leverage points connecting redox balance to immune modulation. Disabling these hubs attenuates persistence and re-sensitizes host defenses, offering complementary routes to reduce pathology in hard-to-oxygenate foci such as mammary tissue microabscesses [40,42,44].

In summary, oxygen availability was associated with marked shifts in the intracellular *S. aureus* transcriptional profile in MAC-T cells with broader category representation under Normal O_2_ and a narrower set of enriched annotations under Reduced O_2_. These observations support the view that oxygen context is an important variable in this model while remaining subject to the experimental limitations noted above.

## 4. Materials and Methods

### 4.1. Bacterial Strain and Growth Preparation

A dominant bovine mastitis clone previously identified in our setting (PFGE type A7; *clfA* subtype Q; identical to the sequenced strain RF122) was used for all experiments. The use of a single, genotyped bovine mastitis clone was intended to minimize the strain-level variation in attachment, invasion, and intracellular transcriptional responses. Stock cultures were stored at −80 °C and revived immediately prior to use to avoid genetic drift. Culture preparation followed the general invasion-assay approaches of Almeida et al. [61] and Bayles et al. [49] with minor adaptations for the present MAC-T system. Briefly, overnight bacterial cultures were prepared in antibiotic- and serum-free invasion medium, pelleted by centrifugation, washed once in sterile phosphate-buffered saline (PBS; pH 7.2), and resuspended to approximately 10^10^ CFU mL^−1^ in invasion medium. Ten-fold serial dilutions were prepared in invasion medium; a 10^2^ dilution was used to inoculate mammary epithelial monolayers at a multiplicity of infection (MOI) of 100. The MOI was selected to enrich for early bacterial entry events under the standardized gentamicin/lysostaphin protection assay used here.

### 4.2. Mammary Epithelial Cell Line and Oxygen Modulation

The MAC-T cell line used in this paper was established and maintained in Dr. Zhao’s laboratory at McGill University, Montreal, Canada. The original MAC-T reports by Huynh et al. and Zhao et al. established and characterized this line as a bovine mammary epithelial cell model [62,63]. MAC-T cells were maintained at 37 °C in 5% CO_2_ in growth medium composed of 44.5% Dulbecco’s Modified Eagle Medium (Gibco BRL, Grand Island, NY, USA), 44.5% RPMI-1640 with L-glutamine (Sigma-Aldrich Ltd., Oakville, ON, Canada), 10% fetal bovine serum (FBS; Invitrogen Canada Inc., Burlington, ON, Canada), and 1× antibiotic/antimycotic solution (Invitrogen Inc.); all media were sterile-filtered through 0.22 µm filters. Cells were seeded at 1 × 10^6^ cells/well and grown for 2–3 days before infection under standard culture conditions. Oxygen modulation was applied during infection and the post-infection intracellular-enrichment period rather than during the 2–3 day pre-infection seeding period. For Reduced O_2_ exposures, 6-well plates were placed in a Modular Incubator Chamber (Model 101; Billups-Rothenberg/Inc., Del Mar, CA, USA) equilibrated at a 5% O_2_ gas-phase setpoint (5% CO_2_, balance N_2_, as recommended by the manufacturer). The chamber was flushed twice with a premixed gas containing 5% O_2_, 5% CO_2_, and 90% N_2_, while both inlet and outlet ports were open. Normal O_2_ exposures were performed in a standard incubator at atmospheric O_2_ with 5% CO_2_. Parallel non-reacted bacterial controls were included for each oxygen condition. These controls were maintained under the same gas condition as the corresponding infection samples but without MAC-T cell exposure. The oxygen terms used throughout this paper refer to the applied chamber/incubator gas-phase exposure conditions: atmospheric O_2_ with 5% CO_2_ for Normal O_2_ and 5% O_2_, 5% CO_2_, and balanced N_2_ for Reduced O_2_.

### 4.3. Contamination Controls and Cell Culture Maintenance

To minimize contamination and preserve culture integrity, all media were sterile filtered (0.22 µm), and a routine cell culture was maintained under standard sterile techniques. The growth medium contained 1× antibiotic/antimycotic, which was removed 16 h before infection when cells were switched to antibiotic- and serum-free invasion medium. After infection, supernatants were removed, monolayers were washed repeatedly, and extracellular bacteria were cleared by sequential lysostaphin and gentamicin treatment as detailed below. The bacterial clone used in all assays was a sequenced and genotyped bovine mastitis isolate (PFGE type A7; *clfA* subtype Q; identical to RF122), and inocula were prepared freshly from adapted cultures for each experiment.

### 4.4. Invasion Assay and Intracellular Enrichment

The invasion medium was defined as cell-culture growth medium without FBS and without antibiotic/antimycotic. Sixteen hours before infection, the cell growth medium was replaced with antibiotic- and serum-free invasion medium. Immediately before infection, monolayers were rinsed once with invasion medium and inoculated with 1 mL of the 10^2^ bacterial dilution (MOI = 100). MOI refers to the initial bacteria-to-epithelial cell inoculum used to standardize infection conditions and does not represent the exact number of bacteria internalized per cell. To synchronize entry, plates were gently swirled for 10 min and incubated for 1 h at 37 °C under the assigned gas condition. Supernatants were removed, and monolayers were washed three times with invasion medium containing lysostaphin (10 µg mL^−1^; Sigma-Aldrich Ltd., Oakville, ON, Canada) to eliminate non-adherent extracellular bacteria. Cells were then incubated with gentamicin (100 µg mL^−1^; Invitrogen Inc.) for a fixed 6 h to eliminate remaining extracellular bacteria while preserving epithelial monolayer integrity. Within each experiment, the gentamicin exposure duration was matched between Normal O_2_ and Reduced O_2_ conditions. RNA was harvested after 1 h of infection followed by 6 h of extracellular-bacteria clearance/intracellular enrichment, corresponding to approximately 7 h after bacterial inoculation. This early harvest point was selected to capture bacterial transcription after host-cell entry and extracellular-bacteria clearance before prolonged intracellular incubation could introduce secondary effects such as toxin accumulation, host-cell damage, apoptosis, or stationary-phase-associated changes. Monolayers were then rinsed with sterile water, scraped using disposable sterile scrapers (Fisher Scientific, Ottawa, ON, Canada), and either processed immediately or stabilized in 5–10 volumes of RNAlater (Applied Biosystems/Ambion Inc., Streetsville, ON, Canada) at −80 °C until RNA extraction. Monolayer integrity was monitored microscopically during the assay; however, a separate quantitative host-cell viability assay was not performed.

### 4.5. RNA Extraction, cDNA Synthesis, and High-Throughput qRT–PCR

Cell lysates were homogenized using a FastPrep instrument (Qbiogene Inc., Carlsbad, CA, USA) for 45 s at maximum speed. The total RNA was isolated using the RiboPure-Bacteria kit (Applied Biosystems/Ambion Inc., Streetsville, ON, Canada) according to the manufacturer’s instructions at the Pathogen Functional Genomics Resource Center, J. Craig Venter Institute (JCVI), Maryland, USA. For each sample, 2 µg RNA was reverse-transcribed with random hexamers using SuperScript III. cDNA was purified on Qiagen (QIAGEN Inc., Germantown, MD, USA) MinElute columns and diluted 1:10 in DEPC-treated water.

Genome-wide expression profiling was performed through the established JCVI/PFGRC high-throughput qRT-PCR expression platform on a Roche LightCycler 480 in 384-well format (Roche Diagnostics, Mannheim, Germany). Gene expression was assayed using 5182 ORF-specific primer sets spanning annotations from *S. aureus* strains COL, Newman, and RF122 and designed by the J. Craig Venter Institute/PFGRC platform. These were target-specific primer pairs rather than housekeeping-gene assays. The working primer concentration was 1.25 µM. Each cDNA sample was combined with 2× SYBR-Green master mix and dispensed into 40 replicate 384-well plates using robotics. The cycling conditions were as follows: 95 °C for 5 min; then 65 cycles of 95 °C for 10 s, 60 °C for 10 s, and 72 °C for 10 s, which was followed by melt-curve analysis. The extended 65-cycle protocol was used as part of the high-throughput platform to maximize the detection of low-abundance bacterial transcripts recovered from host-cell invasion samples with the melt-curve inspection used to support specificity. A full primer-pair catalogue assay/locus identifier, source genome, product annotation, BioQT categories and subcategories were available from the JCVI/PFGRC server platform records. Matched non-reacted bacterial controls were included for each oxygen condition and were used as the reference condition for expression calling. These controls were bacteria processed under the same gas condition without MAC-T cell exposure; they were not housekeeping genes, no-template controls, or non-amplification controls. Absent loci that showed no data were not included in comparative differential expression testing and were reported as absent/not detected rather than treated as low-expression or downregulated features. Differential expression analyses were therefore restricted to loci present in the study strain and with a measurable signal above the background.

### 4.6. Normalization and Expression Calling

For each qRT-PCR plate, technical duplicates were averaged before normalization. To normalize by whole-dataset averaging, first, plate-level mean centering was performed by subtracting the mean Cp value of each 384-well plate from every Cp value on that plate. This step was used to reduce the non-biological plate-to-plate and loading variation across the high-throughput qRT-PCR format. Second, for each locus and biological replicate, the matched-control Cp difference was calculated by subtracting the normalized Cp value of the matched non-reacted bacterial control from the normalized Cp value measured in the corresponding MAC-T infection sample under the same oxygen condition. Thus, Normal O_2_ infection samples were compared with non-reacted bacterial controls maintained under Normal O_2_, and Reduced O_2_ infection samples were compared with non-reacted bacterial controls maintained under Reduced O_2_. The non-reacted controls were bacterial reference cultures and were not housekeeping genes. The direction of change was assigned from the matched-control Cp difference with lower Cp in the infection sample indicating higher transcript abundance and higher Cp indicating lower transcript abundance. A locus was called differentially expressed only when the absolute Cp difference exceeded both (i) the maximal Cp variance observed between technical duplicates across the experiments and (ii) 2 Cp cycles, corresponding to an approximately >4-fold difference, assuming near-doubling amplification per cycle. These calculations were performed separately for each biological replicate experiment using the same workflow and exposure definitions for both oxygen conditions. Loci were retained only when they satisfied the calling criteria consistently across experiments rather than by treating absent or variable replicate values as differential expressions. Loci meeting these criteria were assigned to one of four direction/condition sets: Normal O_2_-upregulated (NU), Normal O_2_-downregulated (ND), Reduced O_2_-upregulated (RU), and Reduced O_2_-downregulated (RD). The between-condition comparison presented in the Results section is therefore a comparison of these matched-control-corrected regulated-locus sets and their functional distributions rather than a direct housekeeping-gene-normalized comparison of gene-of-interest output to housekeeping-gene output.

### 4.7. Functional Annotation and Role Mapping

Normalized locus lists were queried in the Biological Role Query Tool (BioQT v1.1.0) hosted at JCVI. BioQT integrates annotations from CMR Cellular Roles, Gene Ontology, PFam/TIGRFAMs, KEGG pathways and EC/KO identifiers. Returned roles and subsystems were harmonized to major functional roles (reported in Table 1) and sub-roles (reported in Table 2). Each locus was assigned to a single curated bin; hypothetical proteins were retained and labeled as “hypothetical” when appropriate. BioQT outputs were exported as tab-delimited files, which provided the basis for the role counts and the product-level summaries presented in the Supplementary Tables. For tabular summaries, “No Data” indicates loci or assay targets that lacked reportable BioQT role assignment or condition-level expression information after the study-strain and signal-above-background filters were applied. These entries were retained in the summary tables for transparency but were not interpreted as low-expression or biologically downregulated loci.

### 4.8. Statistical Analysis

Three independent infection experiments (biological triplicates) were performed for each oxygen condition with matched non-reacted bacterial controls in each experiment. Technical duplicates were used on each qRT-PCR expression plate as described above. Thus, duplicate values refer only to plate-level technical duplication, whereas the infection experiments themselves were conducted in biological triplicates. Each gene locus was treated as a single analytical unit. In cases of duplicate annotations across source genomes, entries were consolidated by the unique locus tag, and the most specific product description was retained for subsequent classification.

The final gene lists were categorized according to their annotated “Main Biological Role,” following the ontology assignments from the original study. For each of the four expression-call sets (Upregulated at Normal O_2_, Downregulated at Normal O_2_, Upregulated at Reduced O_2_, and Downregulated at Reduced O_2_), the frequency of genes per functional category was tabulated. The within-condition proportion was calculated as (count divided by total number of genes in that condition) × 100 to generate the summary percentages presented in Table 1 and Table 2 and illustrated in Figure 1, Figure 2, Figure 3 and Figure 4. These figure axes therefore show functional-category percentages within each expression-call set and are not housekeeping-gene-normalized expression y-axes.

To illustrate key functional signatures, representative genes involved in virulence, regulation, and metabolism were selected from the comprehensive gene lists in the Supplementary Tables. Their expression-call status under Normal versus Reduced O_2_ was then compiled to highlight oxygen-associated transcriptional patterns (Table 3). Table 3 now spells out the fold criterion for each direction of change: “Upregulated (>4-fold)” and “Downregulated (>4-fold)” indicate that the locus exceeded the >2 Cp-cycle threshold relative to the matched non-reacted bacterial control for that oxygen condition and also exceeded the duplicate-variance criterion. “Not significant” indicates that the locus did not satisfy both criteria. The analysis presented is descriptive, focusing on the categorization and quantification of the differentially expressed gene sets. Because the platform was analyzed using predefined Cp-difference thresholds rather than a genome-wide RNA-seq count model, the primary reported criterion was a reproducible direction of change exceeding the duplicate-variance threshold and >2 Cp cycles (approximately >4-fold). No additional inferential *p*-value model was applied to the functional-category summaries; however, the reported functional patterns are supported by reproducible locus-level expression calls that met the same predefined threshold across independent experiments.

## 5. Conclusions

Our findings support a model in which oxygen availability is associated with a transport/regulator-rich pattern under Normal O_2_ and a more concentrated toxin/iron/cation-associated pattern under Reduced O_2_, while a shared set of cytolysin/leukocidin-related loci remains represented in both conditions. Because this was a descriptive transcriptional study and no independent orthogonal validation assay was added for selected loci, this model should be viewed as a conservative framework for future targeted experiments rather than definitive proof of a complete oxygen-driven virulence program.

## Figures and Tables

**Table 1:** Functional distribution of loci differentially regulated in *S. aureus* under Normal O_2_ relative to the matched non-reacted Normal O_2_ bacterial control.

Main Biological Role	Upregulated LOCI (*n*)	Percentage of Total (*n* = 211)	Downregulated Loci (*n*)	Percentage of Total (*n* = 99)
Cellular processes	37	17.50%	5	5.10%
Hypothetical proteins	35	16.60%	12	12.10%
Transport and binding proteins	24	11.40%	24	24.20%
Cell envelope	16	7.60%	18	18.20%
Regulatory functions	17	8.10%	3	3.00%
Energy metabolism	16	7.60%	7	7.10%
Unknown function	13	6.20%	3	3.00%
Purines, pyrimidines, etc.	10	4.70%	0	0.00%
DNA metabolism	8	3.80%	1	1.00%
No data	35	16.60%	13	13.10%
**Other categories**	0	0.00%	13	13.10%
**TOTAL**	211	100%	99	100%

**Table 2:** Functional distribution of loci differentially regulated in *S. aureus* under Reduced O_2_ relative to the matched non-reacted Reduced O_2_ bacterial control.

Main Biological Role	Upregulated Loci (*n*)	Percentage of Total (*n* = 53)	Downregulated Loci (*n*)	Percentage of Total (*n* = 35)
Cellular processes	23	43.40%	3	8.60%
Hypothetical proteins	3	5.70%	9	25.70%
Unknown function	5	9.40%	3	8.60%
Cell envelope	5	9.40%	2	5.70%
Transport and binding proteins	4	7.50%	2	5.70%
Energy metabolism	5	9.40%	2	5.70%
Purines, pyrimidines, etc.	0	0.00%	6	17.10%
DNA metabolism	1	1.90%	3	8.60%
No data	4	7.50%	0	0.00%
Other categories	3	5.70%	5	14.30%
TOTAL	53	100%	35	100%

**Table 3:** Key virulence- and persistence-associated loci regulated under Normal and Reduced O_2_. Loci are listed according to the matched-control Cp differential-expression criteria defined in the Methods section. “Upregulated (>4-fold)” and “downregulated (>4-fold)” indicate reproducible normalized Cp differences exceeding both the maximal technical-duplicate Cp variance and >2 Cp cycles, corresponding to an approximately >4-fold difference in the indicated direction relative to the matched non-reacted bacterial control for the same oxygen condition. “Not significant” indicates that the locus did not meet both criteria under that condition.

Main Role	Sub-Role	Gene/Product	Locus	Normal O_2_ Response vs. Matched Control	Reduced O_2_ Response vs. Matched Control
Core Virulence Gene Set	Pathogenesis	α-hemolysin (*hly*)	SACOL1173	Upregulated (>4-fold)	Upregulated (>4-fold)
		Cysteine protease (*sspB*)	SACOL1970	Upregulated (>4-fold)	Upregulated (>4-fold)
		δ-hemolysin (*hld*)	SACOL2022	Upregulated (>4-fold)	Upregulated (>4-fold)
		Leukocidin/Aerolysin	SACOL2004/6	Upregulated (>4-fold)	Upregulated (>4-fold)
Normal O_2_ Gene Set	Pathogenesis	Staphylococcal accessory regulator (*sarU*)	SACOL2507	Upregulated (>4-fold)	Not significant (<4-fold or failed duplicate-variance criterion)
		AgrD quorum sensing peptide	SACOL2024	Upregulated (>4-fold)	Not significant (<4-fold or failed duplicate-variance criterion)
	Energy metabolism	Lactate dehydrogenase 1 (*ldh1*)	SACOL0222	Upregulated (>4-fold)	Not significant (<4-fold or failed duplicate-variance criterion)
		Pyruvate formate-lyase (*pflA/B*)	SACOL0204/5	Upregulated (>4-fold)	Not significant (<4-fold or failed duplicate-variance criterion)
Reduced O_2_ Gene Set	Cell envelope	Iron-regulated determinant A (*isdA*)	SACOL1140	Not significant (<4-fold or failed duplicate-variance criterion)	Upregulated (>4-fold)
		Iron-regulated determinant C (*isdC*)	SACOL1141	Not significant (<4-fold or failed duplicate-variance criterion)	Upregulated (>4-fold)
	Unknown function	Fibrinogen-binding protein	SACOL1164	Not significant (<4-fold or failed duplicate-variance criterion)	Upregulated (>4-fold)
Metabolic Suppression	Cell division	Cell division protein (*ftsZ*)	SACOL1199	Not significant (<4-fold or failed duplicate-variance criterion)	Downregulated (>4-fold)
	Purine/Pyrimidine	Pyrimidine biosynthesis (*pyrC*, *pyrF*)	SACOL1213/16	Not significant (<4-fold or failed duplicate-variance criterion)	Downregulated (>4-fold)
	Regulatory	Transcriptional regulator (*GntR*)	SACOL1997	Not significant (<4-fold or failed duplicate-variance criterion)	Downregulated (>4-fold)
Metabolic Switching	Energy metabolism	Lactate dehydrogenase 2 (*ldh2*)	SACOL2618	Downregulated (>4-fold)	Not significant (<4-fold or failed duplicate-variance criterion)
	Cell envelope	Murein biosynthesis (*mraY*)	SACOL1195	Upregulated (>4-fold)	Downregulated (>4-fold)

**Table 4:** Summary of shared and condition-associated gene regulation based on matched-control-corrected oxygen-condition expression calls.

Regulatory Category	Number of Loci	Key Gene Examples (Locus and Product)
Upregulated Genes		
Shared Upregulated Core (Upregulated in Both Conditions)	14	SACOL1153, SACOL1173 (*hly*, α-hemolysin), SACOL1182, SACOL1186 (Phenol-soluble modulin), SACOL1187 (Phenol-soluble modulin), SACOL1220 (Fibrinogen-binding protein), SACOL1948, SACOL1954, SACOL1970 (*sspB*, V8 protease), SACOL2002, SACOL2004 (Leukocidin), SACOL2006 (Leukocidin), SACOL2011, SACOL2022 (*hld*, δ-hemolysin)
Normal O_2_-Specific Upregulated Gene Set (Upregulated Only in Normal O_2_)	161	Supplementary Table S1
Reduced O_2_-Specific Upregulated Gene Set (Upregulated Only in Reduced O_2_)	19	Supplementary Table S3
**Downregulated Genes**		
Shared Downregulated Locus (Downregulated in Both Conditions)	1	SACOL1150 (Cell division protein *FtsQ*)
Normal O_2_-Specific Suppression (Downregulated Only in Normal O_2_)	85	Supplementary Table S2
Reduced O_2_-Specific Suppression (Downregulated Only in Reduced O_2_)	34	Supplementary Table S4

## Data Availability

The original contributions presented in this study are included in the article/Supplementary Materials. Further inquiries can be directed to the corresponding author.

## Supplementary Materials

The following supporting information can be downloaded at https://www.mdpi.com/article/10.3390/ijms27104591/s1.
