# Supplementary material for: Reduced Oxygen Condition Is Associated with Genome-Wide Expression Changes in Mastitis-Lineage Staphylococcus aureus During In Vitro Invasion into a Mammary Cell Line
Source: Int J Mol Sci. 2026 May 20;27(10):4591. doi: 10.3390/ijms27104591 (PMC13206821; doi:10.3390/ijms27104591)
Supplement: Supplementary file 1 [file ijms-27-04591-s001.zip › ijms-4244514-supplementary.pdf]

S. aureus upregulated genes at normal O_2 (Supplementary Table S1)

	Main role*	MC
1	No Data	36
2	No Data	36
3	No Data	36
4	No Data	36
5	No Data	36
6	No Data	36
7	No Data	36
8	No Data	36
9	No Data	36
10	No Data	36
11	No Data	36
12	No Data	36
13	No Data	36
14	No Data	36
15	No Data	36
16	No Data	36
17	No Data	36
18	No Data	36
19	No Data	36
20	No Data	36
21	No Data	36
22	No Data	36
23	No Data	36
24	No Data	36
25	No Data	36
26	No Data	36
27	No Data	36
28	No Data	36
29	No Data	36
30	No Data	36
31	No Data	36
32	No Data	36
33	No Data	36
34	No Data	36
35	No Data	36
36	No Data	34
37	Hypothetical proteins	34
38	Hypothetical proteins	34
39	Hypothetical proteins	34
40	Hypothetical proteins	34
41	Hypothetical proteins	34
42	Hypothetical proteins	34
43	Hypothetical proteins	34
44	Hypothetical proteins	34
45	Hypothetical proteins	34

46	Hypothetical proteins	34
47	Hypothetical proteins	34
48	Hypothetical proteins	34
49	Hypothetical proteins	34
50	Hypothetical proteins	34
51	Hypothetical proteins	34
52	Hypothetical proteins	34
53	Hypothetical proteins	34
54	Hypothetical proteins	34
55	Hypothetical proteins	34
56	Hypothetical proteins	34
57	Hypothetical proteins	34
58	Hypothetical proteins	34
59	Hypothetical proteins	34
60	Hypothetical proteins	34
61	Hypothetical proteins	34
62	Hypothetical proteins	34
63	Hypothetical proteins	34
64	Hypothetical proteins	34
65	Hypothetical proteins	34
66	Hypothetical proteins	34
67	Hypothetical proteins	34
68	Hypothetical proteins	34
69	Hypothetical proteins	34
70	Hypothetical proteins	34
71	Cellular processes	24
72	Cellular processes	24
73	Cellular processes	24
74	Cellular processes	24
75	Cellular processes	24
76	Cellular processes	24
77	Cellular processes	24
78	Cellular processes	24
79	Cellular processes	24
80	Cellular processes	24
81	Cellular processes	24
82	Cellular processes	24
83	Cellular processes	24
84	Cellular processes	24
85	Cellular processes	24
86	Cellular processes	24
87	Cellular processes	24
88	Cellular processes	24
89	Cellular processes	24
90	Cellular processes	24
91	Cellular processes	24
92	Cellular processes	24
93	Cellular processes	24

94	Cellular processes	24
95	Cellular processes	24
96	Cellular processes	24
97	Cellular processes	24
98	Cellular processes	24
99	Cellular processes	24
100	Cellular processes	24
101	Cellular processes	24
102	Cellular processes	24
103	Cellular processes	24
104	Cellular processes	24
105	Cellular processes	24
106	Cellular processes	24
107	Cellular processes	24
108	Transport and binding	24
109	Transport and binding	24
110	Transport and binding	24
111	Transport and binding	24
112	Transport and binding	24
113	Transport and binding	24
114	Transport and binding	24
115	Transport and binding	24
116	Transport and binding	24
117	Transport and binding	24
118	Transport and binding	24
119	Transport and binding	24
120	Transport and binding	24
121	Transport and binding	24
122	Transport and binding	24
123	Transport and binding	24
124	Transport and binding	24
125	Transport and binding	24
126	Transport and binding	24
127	Transport and binding	24
128	Transport and binding	24
129	Transport and binding	24
130	Transport and binding	24
131	Transport and binding	24
132	Cell envelope	16
133	Cell envelope	16
134	Cell envelope	16
135	Cell envelope	16
136	Cell envelope	16
137	Cell envelope	16
138	Cell envelope	16
139	Cell envelope	16
140	Cell envelope	16
141	Cell envelope	16

142	Cell envelope	16
143	Cell envelope	16
144	Cell envelope	16
145	Cell envelope	16
146	Cell envelope	16
147	Cell envelope	16
148	Energy metabolism	14
149	Energy metabolism	14
150	Energy metabolism	14
151	Energy metabolism	14
152	Energy metabolism	14
153	Energy metabolism	14
154	Energy metabolism	14
155	Energy metabolism	14
156	Energy metabolism	14
157	Energy metabolism	14
158	Energy metabolism	14
159	Energy metabolism	14
160	Energy metabolism	14
161	Energy metabolism	14
162	Energy metabolism	14
163	Energy metabolism	14
164	Regulatory functions	14
165	Regulatory functions	14
166	Regulatory functions	14
167	Regulatory functions	14
168	Regulatory functions	14
169	Regulatory functions	14
170	Regulatory functions	14
171	Regulatory functions	14
172	Regulatory functions	14
173	Regulatory functions	14
174	Regulatory functions	14
175	Regulatory functions	14
176	Regulatory functions	14
177	Regulatory functions	14
178	Regulatory functions	14
179	Regulatory functions	14
180	Regulatory functions	14
181	Regulatory functions	14
182	Unknown function	13
183	Unknown function	13
184	Unknown function	13
185	Unknown function	13
186	Unknown function	13
187	Unknown function	13
188	Unknown function	13
189	Unknown function	13

190	Unknown function	13
191	Unknown function	13
192	Unknown function	13
193	Unknown function	13
194	Unknown function	13
195	Purines, pyrimidines, nucleosides, nucleotides tidtinucleotides	10
196	Purines, pyrimidines, nucleosides, nucleotides	10
197	Purines, pyrimidines, nucleosides, nucleotides	10
198	Purines, pyrimidines, nucleosides, nucleotides	10
199	Purines, pyrimidines, nucleosides, nucleotides	10
200	Purines, pyrimidines, nucleosides, nucleotides	10
201	Purines, pyrimidines, nucleosides, nucleotides	10
202	Purines, pyrimidines, nucleosides, nucleotides	10
203	Purines, pyrimidines, nucleosides, nucleotides	10
204	Purines, pyrimidines, nucleosides, nucleotides	10
205	DNA metabolism	8
206	DNA metabolism	8
207	DNA metabolism	8
208	DNA metabolism	8
209	DNA metabolism	8
210	DNA metabolism	8
211	DNA metabolism	8

*MainBiological Role; #Main role-count; Sub-role count; §Gene product; ¶Strain

Sub-role#	SRC	Product
No Data	36	
No Data	36	
No Data	36	
No Data	36	
No Data	36	
No Data	36	
No Data	36	
No Data	36	
No Data	36	
No Data	36	
No Data	36	
No Data	36	
No Data	36	
No Data	36	
No Data	36	
No Data	36	
No Data	36	
No Data	36	
No Data	36	
No Data	36	
No Data	36	
No Data	36	
No Data	36	
No Data	36	
No Data	36	
No Data	36	
No Data	36	
No Data	36	
No Data	36	
No Data	36	
No Data	36	
No Data	36	
No Data	36	
No Data	36	
No Data	36	
No Data	36	
Conserved	31	
Conserved	31	
Conserved	31	
Conserved	31	
Conserved	31	
Conserved	31	
Conserved	31	
Conserved	31	
Conserved	31	

Conserved	31	
Conserved	31	
Conserved	31	
Conserved	31	
Conserved	31	
Conserved	31	
Conserved	31	
Conserved	31	
Conserved	31	
Conserved	31	
Conserved	31	
Conserved	31	
Conserved	31	
Conserved	31	
Conserved	31	
Conserved	31	
Conserved	31	
Conserved	31	
Conserved	31	
Conserved	31	
Conserved	31	
Conserved	31	
Domain	3	
Domain	3	
Domain	3	
Detoxification	1	pnbA, hydrolyse b-lactam PNB
Adaptations to atypical conditions	2	betA
Adaptations to atypical conditions	2	betB
Cell division	1	
Pathogenesis	15	agrA
Pathogenesis	15	agrD
Pathogenesis	15	putative
Pathogenesis	15	putative
Pathogenesis	15	sarU
Pathogenesis	15	sarU
Pathogenesis	15	sspB2
Pathogenesis	15	hld
Pathogenesis	15	hld
Pathogenesis	15	putative
Pathogenesis	15	putative
Pathogenesis	15	hlY
Pathogenesis	15	hlY
Pathogenesis	15	Aerolysis/leukocidin family
Pathogenesis	15	Aerolysis/leukocidin family
Cell adhesion	2	camS, hypothetical protein
Cell adhesion	2	camS, hypothetical protein
Other	2	camS, hypothetical protein
Other	2	camS, hypothetical protein

Toxin production and resistance	15	vraS,
Toxin production and resistance	15	superantigen-like protein
Toxin production and resistance	15	set11, superantigen-like protein
Toxin production and resistance	15	exfoliative toxin, putative
Toxin production and resistance	15	exfoliative toxin, putative
Toxin production and resistance	15	sarU
Toxin production and resistance	15	sarU
Toxin production and resistance	15	hld
Toxin production and resistance	15	hld
Toxin production and resistance	15	Leukocidin, putative
Toxin production and resistance	15	hlY, a-hemolysin precursor
Toxin production and resistance	15	hlY, a-hemolysin precursor
Toxin production and resistance	15	Aerolysin/leukocidi family
Toxin production and resistance	15	Aerolysin/leukocidi family protein
Amino acids, peptides and amines	12	aa ABC transporter, ATP-binding
Amino acids, peptides and amines	12	Oligo peptide, ATP-bindg protein
Amino acids, peptides and amines	12	aa ABC transporter, permease
Amino acids, peptides and amines	12	Oligo pep transport system permease
Amino acids, peptides and amines	12	Oligo peptide, ATP-bindg protein
Amino acids, peptides and amines	12	Oligo peptide, ATP-bindg protein
Amino acids, peptides and amines	12	ABC transporter permease, Putative
Amino acids, peptides and amines	12	ABC transporter permease, Putative
Amino acids, peptides and amines	12	aa ABC transporter, ATP-binding
Amino acids, peptides and amines	12	ABC transporter permease,
Amino acids, peptides and amines	12	aa ABC transporter, ATP-bindg
Amino acids, peptides and amines	12	
Carbohydrates, organic alcohols, acids	2	PTS system, IIBC components
Carbohydrates, organic alcohols, acids	2	citrate transporter, permease
Other	2	multidrug resistance protein
Other	2	MmpL efflux pump, putative
Nucleosides, purines and pyrimidines	2	pbuX, xanthine permease
Nucleosides, purines and pyrimidines	2	uraA, uracil permease
Unknown substrate	3	aa ABC transporter, ATP-binding
Unknown substrate	3	Hypothetical protein
Unknown substrate	3	transporter, putative
Cations and iron carrying compounds	3	copper-translocating P-type ATPase
Cations and iron carrying compounds	3	sodium transport family protein
Cations and iron carrying compounds	3	
Surface structures	1	
Other	11	
Other	11	
Other	11	
Other	11	
Other	11	
Other	11	
Other	11	
Other	11	
Other	11	

Other	11	
Other	11	
Biosynth & degradn of polysach & lipopolysacharides	1	
Biosyn&degradation of mureines and peptidoglycans	3	
Biosyn&degradation of mureines and peptidoglycans	3	mraY
Biosyn&degradation of mureines and peptidoglycans	3	murD
Glycolysis/gluconeogenesis	4	fructose-bisphosphatase
Glycolysis/gluconeogenesis	4	ldh1, L-lactate dehydrogenase
Glycolysis/gluconeogenesis	4	ldh1, L-lactate dehydrogenase
Glycolysis/gluconeogenesis	4	fdaB, fructose-1,6-bi-p aldolase
Fermentation	3	aldA2, aldehyde deHase
Fermentation	3	acetaldehyde-CoA/alco deHase
Fermentation	3	budA2
Anaerobic	4	pflA
Anaerobic	4	pflB, formate acetyltransferase
Anaerobic	4	ldh1, L-lactate dehydrogenase
Anaerobic	4	L-lactate dehydrogenase
Electron transport	1	acpD, azoreductase
Amino acids and amines	4	sdaAB, L-serine dehydratase
Amino acids and amines	4	
Amino acids and amines	4	arcC1, carbamate kinase
Amino acids and amines	4	arcB1
DNA interactions	8	MerR regulor
DNA interactions	8	AraC
DNA interactions	8	AraC
DNA interactions	8	RpiR
DNA interactions	8	sarU,
DNA interactions	8	sarU
DNA interactions	8	GntR transcriptional regulor
DNA interactions	8	TetR transcriptional regulor
RNA interactions	1	pyrR
Other	4	agrA
Other	4	agrD
Other	4	sarU
Other	4	sarU
Protein interactions	5	vraS
Protein interactions	5	yycG
Protein interactions	5	AraC
Protein interactions	5	AraC
Protein interactions	5	
Enzymes of unknown specificity	3	dehydrogenase/reductas
Enzymes of unknown specificity	3	exonuclease
Enzymes of unknown specificity	3	alpha/beta hydrolase
General	10	hypothetical
General	10	murQ
General	10	glyoxalase family
General	10	yycI, hypothetical
General	10	

General	10	hypothetical protein
General	10	sensor histidine kinase
General	10	guanylate kinase
General	10	(phenol sol modulin)
General	10	(phenol sol modulin)
Salvage of nucleosides and nucleotides	1	
Pyrimidine ribonucleotide biosynthesis	4	
Pyrimidine ribonucleotide biosynthesis	4	
Pyrimidine ribonucleotide biosynthesis	4	
Pyrimidine ribonucleotide biosynthesis	4	
Purine ribonucleotide biosynthesis	3	
Purine ribonucleotide biosynthesis	3	
Purine ribonucleotide biosynthesis	3	
2'-Deoxyribonucleotide metabolism	2	
2'-Deoxyribonucleotide metabolism	2	
DNA replication, recombination, repair	8	
DNA replication, recombination, repair	8	
DNA replication, recombination, repair	8	
DNA replication, recombination, repair	8	
DNA replication, recombination, repair	8	
DNA replication, recombination, repair	8	
DNA replication, recombination, repair	8	

in name used for primer

**Locus§**	**Org. descr.¶**
SACOL2502	S.aureus COL
SACOL0500	S.aureus COL
SACOL2454	S.aureus COL
SACOL2466	S.aureus COL
SAB2312	S.aureus RF122
SAB0740	S.aureus RF122
SAB2223	S.aureus RF122
SACOL2580	S.aureus COL
SACOL1165	S.aureus COL
SACOL2503	S.aureus COL
SACOL0216	S.aureus COL
SAB1933	S.aureus RF122
SACOL1948	S.aureus COL
SACOL0532	S.aureus COL
SAB1772	S.aureus RF122
SACOL0465	S.aureus COL
SACOL0463	S.aureus COL
SACOL0208	S.aureus COL
Locus Not CMR	SACOL2554.1
SACOL1971	S.aureus COL
SACOL2470	S.aureus COL
SAB2246c	S.aureus RF122
SAB1890	S.aureus RF122
SAB0334c	S.aureus RF122
SACOL1949	S.aureus COL
SACOL2480	S.aureus COL
SACOL2543	S.aureus COL
SAB1238c	S.aureus RF122
SAB0166	S.aureus RF122
SAB1888c	S.aureus RF122
SACOL2495	S.aureus COL
SAB0206	S.aureus RF122
SAB1771	S.aureus RF122
SAB2360	S.aureus RF122
SAB0641c	S.aureus RF122
SACOL2603	S.aureus COL
SACOL1989	S.aureus COL
SACOL1190	S.aureus COL
SAB0745	S.aureus RF122
SACOL2020	S.aureus COL
SACOL0511	S.aureus COL
SACOL1999	S.aureus COL
NWMN_2555	S.aureus NM
SACOL0512	S.aureus COL
SACOL0166	S.aureus COL

SAB1177	S.aureus RF122
SAB0141	S.aureus RF122
SACOL0443	S.aureus COL
SACOL1201	S.aureus COL
SACOL0530	S.aureus COL
SACOL0499	S.aureus COL
SAB0351	S.aureus RF122
SACOL1975	S.aureus COL
SAB0136c	S.aureus RF122
SACOL1135	S.aureus COL
SACOL1998	S.aureus COL
SACOL2041	S.aureus COL
SACOL2600	S.aureus COL
SACOL2478	S.aureus COL
SACOL2491	S.aureus COL
SACOL2477	S.aureus COL
SAB1647c	S.aureus RF122
SAB1232	S.aureus RF122
SAB1710c	S.aureus RF122
SAB0900	S.aureus RF122
SACOL2479	S.aureus COL
SACOL2609	S.aureus COL
SACOL0207	S.aureus COL
SACOL2599	S.aureus COL
SAB1908c	S.aureus RF122
SACOL2459	S.aureus COL
SACOL2627	S.aureus COL
SACOL2628	S.aureus COL
SACOL1193	S.aureus COL
SACOL2026	S.aureus COL
SACOL2024	S.aureus COL
SACOL1184	S.aureus COL
SACOL1184	S.aureus COL
SACOL2507	S.aureus COL
SACOL2507	S.aureus COL
SACOL1970	S.aureus COL
SACOL2022	S.aureus COL
SACOL2022	S.aureus COL
SACOL2004	S.aureus COL
SACOL2004	S.aureus COL
SACOL1173	S.aureus COL
SACOL1173	S.aureus COL
SACOL2006	S.aureus COL
SACOL2006	S.aureus COL
SACOL1964	S.aureus COL
SACOL1964	S.aureus COL
SACOL1964	S.aureus COL
SACOL1964	S.aureus COL

SACOL1943	S.aureus COL
SACOL0470	S.aureus COL
SAB0376	S.aureus RF122
SACOL1184	S.aureus COL
SACOL1184	S.aureus COL
SACOL2507	S.aureus COL
SACOL2507	S.aureus COL
SACOL2022	S.aureus COL
SACOL2022	S.aureus COL
SACOL2004	S.aureus COL
SACOL1173	S.aureus COL
SACOL1173	S.aureus COL
SACOL2006	S.aureus COL
SACOL2006	S.aureus COL
SACOL2453	S.aureus COL
SAB0861	S.aureus COL
SACOL2452	S.aureus COL
SAB0857	S.aureus RF122
SAB0855	S.aureus RF122
SAB0860	S.aureus RF122
SACOL2475	S.aureus COL
SACOL2476	S.aureus COL
SACOL2473	S.aureus COL
SACOL2474	S.aureus COL
SACOL2472	S.aureus COL
SAB0862	S.aureus RF122
SACOL0178	S.aureus COL
SACOL2636	S.aureus COL
SAB1892c	S.aureus RF122
SACOL2566	S.aureus COL
SACOL0459	S.aureus COL
SACOL1211	S.aureus COL
SACOL2525	S.aureus COL
SAB0137	S.aureus RF122
SACOL2471	S.aureus COL
SACOL2572	S.aureus COL
SACOL2011	S.aureus COL
SACOL0203	S.aureus COL
SAB0387	S.aureus RF122
SAB0140	S.aureus RF122
SACOL2554	S.aureus COL
SACOL2582	S.aureus COL
SACOL0203	S.aureus COL
SACOL2526	S.aureus COL
SACOL2520	S.aureus COL
SACOL2476	S.aureus COL
SAB2368c	S.aureus RF122
SACOL2002	S.aureus COL

SAB0300	S.aureus RF122
SACOL2566	S.aureus COL
SACOL2508	S.aureus COL
SACOL1951	S.aureus COL
SACOL1195	S.aureus COL
SACOL1196	S.aureus COL
SAB2390	S.aureus RF122
SACOL0222	S.aureus COL
SACOL0222	S.aureus COL
SACOL2622	S.aureus COL
SACOL1984	S.aureus COL
SACOL0135	S.aureus COL
SACOL2617	S.aureus COL
SACOL0205	S.aureus COL
SACOL0204	S.aureus COL
SACOL0222	S.aureus COL
SACOL0222	S.aureus COL
SACOL0190	S.aureus COL
SACOL2545	S.aureus COL
SACOL2569	S.aureus COL
SACOL1182	S.aureus COL
SACOL1181	S.aureus COL
SACOL2517	S.aureus COL
SACOL0201	S.aureus COL
SACOL0201	S.aureus COL
SACOL0179	S.aureus COL
SACOL2507	S.aureus COL
SACOL2507	S.aureus COL
SACOL0518	S.aureus COL
SACOL2610	S.aureus COL
SACOL1210	S.aureus COL
SACOL2026	S.aureus COL
SACOL2024	S.aureus COL
SACOL2507	S.aureus COL
SACOL2507	S.aureus COL
SACOL1943	S.aureus COL
SACOL0020	S.aureus COL
SACOL0201	S.aureus COL
SACOL0201	S.aureus COL
SACOL1232	S.aureus COL
SACOL2488	S.aureus COL
SACOL1954	S.aureus COL
SACOL2597	S.aureus COL
SACOL0014	S.aureus COL
SACOL0177	S.aureus COL
SACOL1207	S.aureus COL
SACOL0022	S.aureus COL
SACOL1967	S.aureus COL

SACOL1162	S.aureus COL
SACOL0202	S.aureus COL
SACOL1220	S.aureus COL
SACOL1187	S.aureus COL
SACOL1186	S.aureus COL
SACOL0458	S.aureus COL
SACOL1213	S.aureus COL
SACOL1216	S.aureus COL
SACOL1217	S.aureus COL
SACOL1214	S.aureus COL
SACOL0460	S.aureus COL
SACOL0018	S.aureus COL
SACOL1969	S.aureus COL
SACOL2634	S.aureus COL
SACOL2635	S.aureus COL
SACOL2500	S.aureus COL
SAB0088	S.aureus RF122
SACOL0004	S.aureus COL
SACOL0526	S.aureus COL
SACOL1153	S.aureus COL
SACOL0441	S.aureus COL
SAB1745c	S.aureus RF122

S. aureus Downregulated genes at normal O_2

Supplementary Table S2

	Main role*	MC
1	Transport and binding proteins	22
2	Transport and binding proteins	22
3	Transport and binding proteins	22
4	Transport and binding proteins	22
5	Transport and binding proteins	22
6	Transport and binding proteins	22
7	Transport and binding proteins	22
8	Transport and binding proteins	22
9	Transport and binding proteins	22
10	Transport and binding proteins	22
11	Transport and binding proteins	22
12	Transport and binding proteins	22
13	Transport and binding proteins	22
14	Transport and binding proteins	22
15	Transport and binding proteins	22
16	Transport and binding proteins	22
17	Transport and binding proteins	22
18	Transport and binding proteins	22
19	Transport and binding proteins	22
20	Transport and binding proteins	22
21	Transport and binding proteins	22
22	Transport and binding proteins	22
23	Transport and binding proteins	22
24	Transport and binding proteins	22
25	Cell envelope	18
26	Cell envelope	18
27	Cell envelope	18
28	Cell envelope	18
29	Cell envelope	18
30	Cell envelope	18
31	Cell envelope	18
32	Cell envelope	18
33	Cell envelope	18
34	Cell envelope	18
35	Cell envelope	18
36	Cell envelope	18
37	Cell envelope	18
38	Cell envelope	18
39	Cell envelope	18
40	Cell envelope	18
41	Cell envelope	18
42	Cell envelope	18
43	No Data	13
44	No Data	13
45	No Data	13

46	No Data	13
47	No Data	13
48	No Data	13
49	No Data	13
50	No Data	13
51	No Data	13
52	No Data	13
53	No Data	13
54	No Data	13
55	No Data	13
56	Hypothetical proteins	12
57	Hypothetical proteins	12
58	Hypothetical proteins	12
59	Hypothetical proteins	12
60	Hypothetical proteins	12
61	Hypothetical proteins	12
62	Hypothetical proteins	12
63	Hypothetical proteins	12
64	Hypothetical proteins	12
65	Hypothetical proteins	12
66	Hypothetical proteins	12
67	Hypothetical proteins	12
68	Energy metabolism	4
69	Energy metabolism	4
70	Energy metabolism	4
71	Energy metabolism	4
72	Energy metabolism	4
73	Energy metabolism	4
74	Cellular processes	4
75	Cellular processes	4
76	Cellular processes	4
77	Cellular processes	4
78	Regulatory functions	3
79	Regulatory functions	3
80	Regulatory functions	3
81	Fatty acd & phospholipd meta.	3
82	Fatty acd & phospholipd meta.	3
83	Fatty acd & phospholipd meta.	3
84	Fatty acd & phospholipd meta.	3
85	Fatty acd & phospholipd meta.	3
86	Fatty acd & phospholipd meta.	3
87	Unknown function	3
88	Unknown function	3
89	Unknown function	3
90	Signal transduction	2
91	Signal transduction	2
92	Biosy cofactrs, pros grps& carirs	2
93	Biosy cofactrs, pros grps& carirs	2

94	Transcription	2
95	Transcription	2
96	DNA metabolism	1
97	Amino acid biosynthesis	1
98	Central intermediary metabolis	1
99	Protein fate	1

*MainBiological Role; #Main role-count; Sub-role count; §Gene product; ¶Strain

Sub-role#	SRC	Product
Amino acids, peptides and amines	4	
Amino acids, peptides and amines	4	
Amino acids, peptides and amines	4	
Amino acids, peptides and amines	4	
Carbohydrates, organic alcohols, and acids	8	
Carbohydrates, organic alcohols, and acids	8	
Carbohydrates, organic alcohols, and acids	8	
Carbohydrates, organic alcohols, and acids	8	
Carbohydrates, organic alcohols, and acids	8	
Carbohydrates, organic alcohols, and acids	8	
Carbohydrates, organic alcohols, and acids	8	
Carbohydrates, organic alcohols, and acids	8	
Anions	1	
Unknown substrate	9	
Unknown substrate	9	
Unknown substrate	9	
Unknown substrate	9	
Unknown substrate	9	
Unknown substrate	9	
Unknown substrate	9	
Unknown substrate	9	
Unknown substrate	9	
Cations and iron carrying compounds	2	
Cations and iron carrying compounds	2	
Other	7	
Other	7	
Other	7	
Other	7	
Other	7	
Other	7	
Other	7	
Biosynth &degradn of surface polysac & lipopolysac	11	
Biosynth &degradn of surface polysac & lipopolysac	11	
Biosynth &degradn of surface polysac & lipopolysac	11	
Biosynth &degradn of surface polysac & lipopolysac	11	
Biosynth &degradn of surface polysac & lipopolysac	11	
Biosynth &degradn of surface polysac & lipopolysac	11	
Biosynth &degradn of surface polysac & lipopolysac	11	
Biosynth &degradn of surface polysac & lipopolysac	11	
Biosynth &degradn of surface polysac & lipopolysac	11	
Biosynth &degradn of surface polysac & lipopolysac	11	
Biosynth &degradn of surface polysac & lipopolysac	11	
No Data	13	
No Data	13	
No Data	13	

No Data	13
No Data	13
No Data	13
No Data	13
No Data	13
No Data	13
No Data	13
No Data	13
No Data	13
No Data	13
Conserved	11
Conserved	11
Conserved	11
Conserved	11
Conserved	11
Conserved	11
Conserved	11
Conserved	11
Conserved	11
Conserved	11
Conserved	11
Domain	1
Glycolysis/gluconeogenesis	2
Glycolysis/gluconeogenesis	2
Fermentation	1
Anaerobic	2
Anaerobic	2
Electron transport	1
Detoxification	2
Detoxification	2
Cell division	1
Toxin production and resistance	1
Other	2
Other	2
Protein interactions	1
Degradation	2
Degradation	2
Biosynthesis	1
Prophage functions	1
Transposon functions	1
Other	1
General	3
General	3
General	3
PTS	2
PTS	2
Glutathione and analogs	1
Other	1

DNA-dependent RNA polymerase	2
DNA-dependent RNA polymerase	2
DNA replication, recombination, and repair	1
Glutamate family	1
Other	1
Degradation of proteins, peptides, & glycopeptides	1

in name used for primer

Locus§	Org. descr.¶
	S.aureus COL
SACOL2632	S.aureus COL
SACOL0171	S.aureus COL
SACOL0010	S.aureus COL
SACOL2441	S.aureus COL
SACOL0193	S.aureus RF122
SAB0782	S.aureus COL
SACOL0194	S.aureus COL
SACOL0192	S.aureus COL
SACOL2552	S.aureus COL
SACOL0195	S.aureus COL
SACOL2514	S.aureus COL
SACOL0136	S.aureus RF122
SAB2283c	S.aureus COL
SACOL2521	S.aureus COL
SACOL0505	S.aureus RF122
SAB2310c	S.aureus COL
SACOL0504	S.aureus COL
SACOL0506	S.aureus RF122
SAB2309c	S.aureus COL
SACOL1979	S.aureus COL
SACOL1979	S.aureus RF122
SAB2407	S.aureus COL
SACOL1979	S.aureus COL
SACOL1979	S.aureus RF122
SAB0392	S.aureus RF122
SAB2220c	S.aureus RF122
SAB1660c	S.aureus COL
SACOL0193	S.aureus RF122
SAB0396	S.aureus RF122
SAB2529c	S.aureus COL
SACOL2443	S.aureus RF122
SAB0901	S.aureus COL
SACOL0146	S.aureus COL
SACOL0144	S.aureus COL
SACOL2578	S.aureus COL
SACOL0143	S.aureus COL
SACOL0149	S.aureus COL
SACOL0141	S.aureus COL
SACOL0145	S.aureus COL
SACOL0147	S.aureus COL
SACOL0148	S.aureus COL
SACOL0136	S.aureus RF122
SAB2177	S.aureus RF122
SAB0826c	S.aureus RF122
SAB0684	S.aureus RF122

SAB1689c	S.aureus NM
NWMN_2520	S.aureus COL
SACOL0219	S.aureus COL
SACOL2567	S.aureus RF122
SAB0929c	S.aureus RF122
SAB1294	S.aureus COL
SACOL2568	S.aureus RF122
SAB0375c	S.aureus RF122
SAB1881c	S.aureus COL
SACOL1225	S.aureus RF122
SAB1375c	S.aureus COL
SACOL2631	S.aureus COL
SACOL0444	S.aureus COL
SACOL0495	S.aureus COL
SACOL0446	S.aureus COL
SACOL0129	S.aureus COL
SACOL2625	S.aureus COL
SACOL2519	S.aureus COL
SACOL2542	S.aureus RF122
SAB0220	S.aureus COL
SACOL2626	S.aureus COL
SACOL0445	S.aureus COL
SACOL2618	S.aureus COL
SACOL2618	S.aureus COL
SACOL0215	S.aureus COL
SACOL2618	S.aureus COL
SACOL2618	S.aureus COL
SACOL0494	S.aureus COL
SACOL2641	S.aureus COL
SACOL0451	S.aureus COL
SACOL1202	S.aureus COL
SACOL2465	S.aureus RF122
SAB1289c	S.aureus COL
SACOL2585	S.aureus COL
SACOL1939	S.aureus COL
SACOL0214	S.aureus COL
SACOL0213	S.aureus COL
SACOL2482	S.aureus RF122
SAB1727c	S.aureus RF122
SAB1167c	S.aureus COL
SACOL2465	S.aureus RF122
SAB1660c	S.aureus COL
SACOL1941	S.aureus COL
SACOL2484	S.aureus RF122
SAB0782	S.aureus COL
SACOL2552	S.aureus COL
SACOL2641	S.aureus COL
SACOL2579	S.aureus RF122

SAB2012c	S.aureus COL
SACOL1222	S.aureus COL
SACOL1150	S.aureus COL
SACOL0514	S.aureus COL
SACOL2577	S.aureus RF122
SAB2566	

S. aureus upregulated genes at reduced O_2 (Supplementary Table S3).

	Main role*	MC
1	Cellular processes	13
2	Cellular processes	13
3	Cellular processes	13
4	Cellular processes	13
5	Cellular processes	13
6	Cellular processes	13
7	Cellular processes	13
8	Cellular processes	13
9	Cellular processes	13
10	Cellular processes	13
11	Cellular processes	13
12	Cellular processes	13
13	Cellular processes	13
14	Cellular processes	13
15	Cellular processes	13
16	Cellular processes	13
17	Cellular processes	13
18	Cellular processes	13
19	Cellular processes	13
20	Cellular processes	13
21	Cellular processes	13
22	Cellular processes	13
23	Cellular processes	13
24	Unknown function	6
25	Unknown function	6
26	Unknown function	6
27	Unknown function	6
28	Unknown function	6
29	Cell envelope	5
30	Cell envelope	5
31	Cell envelope	5
32	Cell envelope	5
33	Cell envelope	5
34	Trans bindg prot	4
35	Trans bindg prot	4
36	Trans bindg prot	4
37	Trans bindg prot	4
38	No Data	4
39	No Data	4
40	No Data	4
41	No Data	4
42	Energ metabolism	3
43	Energymetabolism	3
44	Energymetabolism	3
45	Energ metabolism	3

46	Energymetabolism	3
47	Hypothetical	3
48	Hypothetical	3
49	Hypothetical	3
50	Protein fate	1
51	F acid, p-lipid met	1
52	DNA metabolism	1
53	Transcription	1

*MainBiological Role; #Main role-count; Sub-role count; §Gene product; ¶Strain

Sub-role#	SRC	Product
Pathogenesis	11	hly, alpha-hemolysin
Pathogenesis	11	hly, alpha-hemolysin
Pathogenesis	11	superantigen-like pt
Pathogenesis	11	superantigen-like pt
Pathogenesis	11	cys ptease precu SspB
Pathogenesis	11	Leukocidin
Pathogenesis	11	Leukocidin
Pathogenesis	11	Aeroly/leuko
Pathogenesis	11	Aeroly/leuko
Pathogenesis	11	hld, delta-hemo
Pathogenesis	11	hld, delta-hemo
Toxin prd& resist	12	Hypothetical
Toxin prd& resist	12	hly, alpha-hemoly
Toxin prd& resist	12	hly, alpha-hemolysin
Toxin prd& resist	12	superantigen-like pt
Toxin prd& resist	12	superantigen-like pt
Toxin prd& resist	12	Leukocidin
Toxin prd& resist	12	Leukocidin
Toxin pd& resist	12	Aeroly/leuko
Toxin prd& resist	12	Aeroly/leuko
Toxin prd& resist	12	TcmP, putative
Toxin prd& resist	12	hld, delta-hemo
Toxin prd& resist	12	hld, delta-hemo
Enzy unkno speci	2	exonuclease
Enzy unkno speci	2	hydrolase
General	4	phenol-soluble modul
General	4	phenol-soluble modul
General	4	fgen bindg-related pt
Other	5	isdA
Other	5	isdC
Other	5	
Other	5	iron-binding prot
Other	5	SdrH, putative
Cations&F- copds	4	iron-binding prot
Cations&F- copds	4	iron-binding prot
Cations&F- copds	4	Na-transport protein
Cations&F- copds	4	Ammonium trans prot
No Data	4	
No Data	4	
No Data	4	
No Data	4	
Electron transport	2	sdhC,succ dehase,TCA
Electron transport	2	sdhC,succ dehase,TCA
TCA cycle	2	sdhC,succ dehase,TCA
TCA cycle	2	sdhC,succ dehase,TCA

Ami acids amines	1	arcC1,carbamate kinase
Conserved	3	ZapA, cell divi protein
Conserved	3	
Conserved	3	
Deg pts, pept, glycopep	1	cys ptease precu SspB
Degradation	1	hlb,phospholipase C
DNA repl, recomb, repair	1	
DNA-dep RNA polyase	1	

in name used for primer

Locus§	Org. descr.¶
	S. aureus COL
SACOL1173	S. aureus COL
SACOL1173	S. aureus COL
SACOL1180	S. aureus COL
SACOL1180	S. aureus COL
SACOL1970	S. aureus COL
SACOL2004	S. aureus COL
SACOL2004	S. aureus COL
SACOL2006	S. aureus COL
SACOL2006	S. aureus COL
SACOL2022	S. aureus COL
SACOL2022	S. aureus COL
SACOL1152	S. aureus COL
SACOL1173	S. aureus COL
SACOL1173	S. aureus COL
SACOL1180	S. aureus COL
SACOL1180	S. aureus COL
SACOL2004	S. aureus COL
SACOL2004	S. aureus COL
SACOL2006	S. aureus COL
SACOL2006	S. aureus COL
SACOL2009	S. aureus COL
SACOL2022	S. aureus COL
SACOL2022	S. aureus COL
SACOL1954	S. aureus COL
SACOL2021	S. aureus COL
SACOL1186	S. aureus COL
SACOL1187	S. aureus COL
SACOL1220	S. aureus COL
SACOL1140	S. aureus COL
SACOL1141	S. aureus COL
SACOL2002	S. aureus COL
SACOL2010	S. aureus COL
SACOL2019	S. aureus COL
SACOL1144	S. aureus COL
SACOL2010	S. aureus COL
SACOL2011	S. aureus COL
SACOL2031	S. aureus COL
SACOL1166	S. aureus COL
SACOL1167	S. aureus COL
SACOL1172	S. aureus COL
SACOL1948	S. aureus COL
SACOL1158	S. aureus COL
SACOL1158	S. aureus COL
SACOL1158	S. aureus COL
SACOL1158	S. aureus COL

SACOL1182	S. aureus COL
SACOL1151	S. aureus COL
SACOL1947	S. aureus COL
SACOL1986	S. aureus COL
SACOL1970	S. aureus COL
SACOL2003	S. aureus COL
SACOL1153	S. aureus COL
SACOL1222	

S. aureus downregulated genes at reduced O_2 (Supplementary Table S4).

	Main role*	MC
1	Hypothetical proteins	9
2	Hypothetical proteins	9
3	Hypothetical proteins	9
4	Hypothetical proteins	9
5	Hypothetical proteins	9
6	Hypothetical proteins	9
7	Hypothetical proteins	9
8	Hypothetical proteins	9
9	Hypothetical proteins	9
10	Purines, pyrimidines, nucleosides &nuctides	6
11	Purines, pyrimidines, nucleosides &nuctides	6
12	Purines, pyrimidines, nucleosides &nuctides	6
13	Purines, pyrimidines, nucleosides &nuctides	6
14	Purines, pyrimidines, nucleosides &nuctides	6
15	Purines, pyrimidines, nucleosides &nuctides	6
16	Unknown function	5
17	Unknown function	5
18	Unknown function	5
19	Cellular processes	3
20	Cellular processes	3
21	Cellular processes	3
22	DNA metabolism	3
23	DNA metabolism	3
24	DNA metabolism	3
25	Protein fate	2
26	Protein fate	2
27	Energy metabolism	2
28	Energy metabolism	2
29	Cell envelope	2
30	Cell envelope	2
31	Transport & binding proteins	2
32	Transport & binding proteins	2
33	Regulatory functions	2
34	Regulatory functions	2
35	Central metabolism	1

*MainBiological Role; #Main role-count; Sub-role count; §Gene product; ¶Strai

Sub-role#	SRC	Product
Conserved	9	hypothetical
Conserved	9	hypothetical
Conserved	9	hypothetical
Conserved	9	hypothetical
Conserved	9	hypothetical
Conserved	9	hypothetical
Conserved	9	hypothetical
Conserved	9	hypothetical
Conserved	9	hypothetical
Pyrimidine biosynthesis	6	pyrB
Pyrimidine biosynthesis	6	pyrC
Pyrimidine biosynthesis	6	carA
Pyrimidine biosynthesis	6	carB
Pyrimidine biosynthesis	6	pyrF
Pyrimidine biosynthesis	6	pyrE,
General	3	fib-bind related
General	3	MraZ, cell division
General	3	
Cell division	3	cell division
Cell division	3	ftsA
Cell division	3	ftsZ
DNA replication, recombination,&repair	3	FtsQ
DNA replication, recombination,&repair	3	MutS2
DNA replication, recombination,&repair	3	ligA, DNAligase
Protein folding& stabilization	2	groEL, chaperonin
Protein folding& stabilization	2	groES,cochaperonin
Sugars	1	fructokinase
Biosynthesis &degrad polysaccharides	1	cscA
Biosynthesis &degrad polysaccharides	2	mraY
Biosynthesis &degrad polysaccharides	2	murD
aa, peptides and amines	1	proline permease
Nucleosides, purines &pyrimidines	1	uraA,uracilpermase
DNA interactions	1	GntR
RNA interactions	1	pyrR
Phosphorus compounds	1	ppaC

in name used for primer

Locus§	Org. descr.¶
SACOL1190	S aureus COL
SACOL1218	S aureus COL
SACOL1226	S aureus COL
SACOL1968	S aureus COL
SACOL1985	S aureus COL
SACOL1987	S aureus COL
SACOL1991	S aureus COL
SACOL1992	S aureus COL
SACOL1993	S aureus COL
SACOL1212	S aureus COL
SACOL1213	S aureus COL
SACOL1214	S aureus COL
SACOL1215	S aureus COL
SACOL1216	S aureus COL
SACOL1217	S aureus COL
SACOL1164	S aureus COL
SACOL1191	S aureus COL
SACOL1204	S aureus COL
SACOL1197	S aureus COL
SACOL1198	S aureus COL
SACOL1199	S aureus COL
SACOL1150	S aureus COL
SACOL1154	S aureus COL
SACOL1965	S aureus COL
SACOL2016	S aureus COL
SACOL2017	S aureus COL
SACOL2028	S aureus COL
SACOL2029	S aureus COL
SACOL1195	S aureus COL
SACOL1196	S aureus COL
SACOL1963	S aureus COL
SACOL1211	S aureus COL
SACOL1997	S aureus COL
SACOL1210	S aureus COL
SACOL1982	S aureus COL
